# The Diguanylate Cyclase SadC Is a Central Player in Gac/Rsm-Mediated Biofilm Formation in Pseudomonas aeruginosa

**DOI:** 10.1128/JB.01850-14

**Published:** 2014-12

**Authors:** Joana A. Moscoso, Tina Jaeger, Martina Valentini, Kailyn Hui, Urs Jenal, Alain Filloux

**Affiliations:** a MRC Centre for Molecular Bacteriology and Infection, Department of Life Sciences, Imperial College London, London, United Kingdom; b Biozentrum of the University of Basel, Basel, Switzerland

## Abstract

Pseudomonas aeruginosa is a Gram-negative opportunistic human pathogen and a threat for immunocompromised and cystic fibrosis patients. It is responsible for acute and chronic infections and can switch between these lifestyles upon taking an informed decision involving complex regulatory networks. The RetS/LadS/Gac/Rsm network and the cyclic-di-GMP (c-di-GMP) signaling pathways are both central to this phenomenon redirecting the P. aeruginosa population toward a biofilm mode of growth, which is associated with chronic infections. While these two pathways were traditionally studied independently from each other, we recently showed that cellular levels of c-di-GMP are increased in the hyperbiofilm *retS* mutant. Here, we have formally established the link between the two networks by showing that the SadC diguanylate cyclase is central to the Gac/Rsm-associated phenotypes, notably, biofilm formation. Importantly, SadC is involved in the signaling that converges onto the RsmA translational repressor either via RetS/LadS or via HptB/HsbR. Although the level of expression of the *sadC* gene does not seem to be impacted by the regulatory cascade, the production of the SadC protein is tightly repressed by RsmA. This adds to the growing complexity of the signaling network associated with c-di-GMP in P. aeruginosa. While this organism possesses more than 40 c-di-GMP-related enzymes, it remains unclear how signaling specificity is maintained within the c-di-GMP network. The finding that SadC but no other diguanylate cyclase is related to the formation of biofilm governed by the Gac/Rsm pathway further contributes to understanding of this insulation mechanism.

## INTRODUCTION

Bacteria adopt different lifestyles in response to the fluctuating conditions that they encounter in the environment. They can form a biofilm, which is a sessile community of bacteria, and they can switch between a motile and a sessile lifestyle (1, 2). In a biofilm, bacteria are engulfed in an extracellular matrix composed of exopolysaccharides, extracellular DNA, and proteins (3–6). This helps protect bacteria from various stresses, harsh antimicrobial treatments, or eradication by the immune system (7). In the case of the opportunistic Gram-negative pathogen Pseudomonas aeruginosa, several exopolysaccharides have been described. Whereas mucoid strains isolated from cystic fibrosis patients overproduce alginate (8), nonmucoid strains, such as PAO1, PA14, or PAK, can produce the Pel exopolysaccharide, a glucose-rich polymer (9–11), and/or the Psl exopolysaccharide, a polymer of a repeating pentamer containing d-mannose, l-rhamnose, and d-glucose (12).

The switch in lifestyle and the development of biofilms are based on informed decisions relayed via complex regulatory networks. This results in the control of various cellular processes at the transcriptional, posttranscriptional, or posttranslational level. In the last decade, key networks, such as the Gac/Rsm pathway or the second messenger, cyclic-di-GMP (c-di-GMP) signaling pathway, have been proven to be central in modulating the transition to biofilms (13, 14). In P. aeruginosa, the Gac/Rsm pathway includes the RetS and LadS sensors that antagonistically impact the activity of the GacS histidine kinase (15–18). RetS can form heterodimers with GacS, thus preventing phosphorylation of the cognate response regulator GacA (19), which otherwise promotes the transcription of two small RNAs, RsmY and RsmZ (20). More recently, it was shown that RetS activity could be counteracted by the PA1611 sensor histidine kinase (21). Ultimately, the small RNAs sequester RsmA, a repressor that inhibits the translation of target mRNAs by binding to single-stranded GGA motifs formed near the ribosome binding site (22–24). Among the more than 500 targets of RsmA are the transcripts of the *pel* and *psl* genes (22, 25). Hence, a *retS* or an *rsmA* mutant has a hyperbiofilm phenotype due to the stimulation of the Gac pathway and the relieved RsmA repression on exopolysaccharide production.

In parallel to the Gac/Rsm pathway, signaling networks mediated by c-di-GMP are also related to the transition to a biofilm (14, 26, 27). This cyclic dinucleotide is ubiquitous among bacteria, with low intracellular levels of c-di-GMP promoting a motile lifestyle and high levels promoting biofilm formation (28). Proteins involved in the synthesis of c-di-GMP harbor a GGDEF domain and are known as diguanylate cyclases (DGCs) (29). Proteins with an EAL or HD-GYP domain are called phosphodiesterases (PDEs) and are involved in c-di-GMP hydrolysis (29).

Recently, it was shown that a *retS* mutant displays elevated levels of c-di-GMP, suggesting a link between the two networks (30). In the present work, we have attempted to pinpoint the molecular basis of this link. We report that the membrane-associated DGC known as SadC (31) is central to the Gac/Rsm pathway and that additional elements, such as SadB, also play a role in the signaling cascade.

## MATERIALS AND METHODS

### Strains, plasmids, and growth conditions.

The bacterial strains, plasmids, and primers used in this study are listed in Table S1 in the supplemental material. Bacteria were cultured at 37°C with shaking in LB medium or M9 minimal medium containing 22 mM glucose, 2 mM MgSO_4_, and 0.1 mM CaCl_2_. Culture media were supplemented with antibiotics at the following concentrations, when appropriate: for Escherichia coli, 50 μg/ml ampicillin (Ap) and 50 μg/ml kanamycin (Km), and for P. aeruginosa, 500 μg/ml carbenicillin (Cb) for selection or 300 μg/ml Cb for maintenance, 2,000 μg/ml streptomycin (Sm) for selection, 150 μg/ml gentamicin (Gm) for selection and 100 μg/ml Gm for maintenance, and 50 μg/ml tetracycline (Tet) for maintenance. A Congo red staining assay was performed at 30°C on tryptone (10 g/liter) agar (1%) plates supplemented with 40 μg/ml Congo red and 20 μg/ml Coomassie brilliant blue. Plates for swimming motility assays were prepared using 0.3% LB agar and incubated at 30°C as described previously (32). Escherichia coli OmniMAX and TOP10 were used for standard genetic manipulations. PCR products were cloned into pCR2.1-TA and subcloned into pBBR1MCS-4 or pKNG101. Transfer of plasmids into P. aeruginosa strains was carried out by triparental mating using the conjugative properties of plasmid pRK2013. Transconjugants were isolated on Pseudomonas isolation agar (Difco) supplemented with the appropriate antibiotics. Deletion mutants were selected in 5% sucrose after 3 days of incubation at room temperature. Mutator fragments were constructed by PCR amplification of upstream and downstream fragments of approximately 500 bp flanking the chromosomal region to be mutated. Deletions were confirmed by sequencing using external primers.

### Biofilm assays.

Visualization of biofilm formation was carried out in 14-ml borosilicate tubes. Briefly, LB (3 ml) or M9 medium supplemented with appropriate antibiotics was inoculated to a final optical density at 600 nm (OD_600_) of 0.1 and incubated at 37°C. Biofilms were stained with 0.1% crystal violet (CV), and tubes were washed with water to remove unbound dye. Quantification of biofilm formation was performed in 24-well polystyrene microtiter plates. LB (1 ml/well) and antibiotics, when appropriate, were inoculated to a final OD_600_ of 0.01. The plates were incubated for 6 h or 14 h at 30°C. Biofilms were stained with 100 μl of CV and washed twice with water before being solubilized in 96% ethanol. CV staining was measured by reading the optical density at 600 nm.

### RNA extraction and qRT-PCR.

Overnight PAK and PAK Δ*retS* cultures were subcultured in LB medium with a starting OD_600_ of 0.1 and incubated at 37°C with shaking for 6 h. Cells were then harvested into RNAlater stabilization solution (Ambion), and RNA was extracted using an RNeasy extraction kit (Qiagen). To remove DNA, a Turbo DNA-free kit (Applied Biosystems) was used, and the RNA was repurified using an RNeasy kit, following the supplier's indications. cDNA was synthesized from 200 ng of RNA template by adding 20 U of Protector RNase inhibitor from Roche, 10 pmol of Pd(N)_6_ random hexamer oligonucleotides from Amersham, and 10 pmol deoxynucleoside triphosphates from Bioline to the reaction mix. Quantitative real-time reverse transcription-PCR (qRT-PCR) was performed on an ABI 7300 real-time PCR system using ABI SYBR green PCR master mix.

### Quantification of c-di-GMP.

The reporter P*cdrA-gfp* plasmid, obtained from Matthew Parsek (33), was introduced into the P. aeruginosa strains of interest by electroporation, and overnight cultures were subcultured in LB medium supplemented with the appropriate antibiotic to a starting OD_600_ of 0.1. After shaking incubation at 37°C for 6 h, 1 ml of culture was harvested and cells were resuspended in 1× phosphate-buffered saline (PBS) before the optical density at 600 nm and fluorescence (excitation, 485 nm; emission, 520 nm) were measured in a black 96-well plate with a see-through bottom (Falcon) using a FLUOstar Optima plate reader (BMG Labtech). Quantifications were performed in triplicate, and data are presented as relative fluorescent units (RFU), which are arbitrary fluorescent units corrected for cell density.

### Construction of chromosomal Flag fusions.

The PAO1 strains encoding DGC proteins with a C-terminal Flag tag were engineered using a strategy previously described (34). Briefly, the Flag fusion constructs were produced by splicing by overhang extension PCR using the primers listed in Table S1 in the supplemental material and contained 500- to 700-bp homologous flanking regions, with the Flag tag positioned in the middle. This construct was ligated into pME3087 between BamHI and HindIII/EcoRI restrictions sites. The resulting vector was then used to introduce the Flag tag fusion by a two-step allelic exchange. Following biparental conjugation (donor S17-1) into the target strain, single crossovers were selected on Tet and restreaked. Cultures from single crossovers were grown overnight in LB medium and diluted 1:100 into fresh medium. After 2 h, 20 μg/ml Tet was added to inhibit the growth of cells that had lost the Tet resistance cassette. After a further hour of growth, 2,000 μg/ml Cb was added to select against growing bacteria. Cultures were grown for a further 4 to 6 h, before cells were harvested by centrifugation, washed once in LB, and used to inoculate an overnight culture. This counterselection was done twice, before plating a dilution series of the final samples onto LB agar. Individual colonies were patched onto LB plates with or without Tet, and Tet-sensitive colonies were tested for Flag insertion by colony PCR.

### Western blots.

Strains with chromosomal Flag-tagged DGCs containing a vector with either *rsmY* or *rsmA* under the control of an inducible promoter were grown overnight, diluted 1:100 in the morning, and grown to an OD_600_ of 1.5. This culture was used to inoculate 20 ml prewarmed LB supplemented with 0.05% Triton X-100. At an OD_600_ of 0.4, 1 mM IPTG (isopropyl-β-d-thiogalactopyranoside) was added for induction of *rsmY* or *rsmA*. After induction, samples were taken every hour. Samples were separated on 12% Tris-HCl gels and blotted onto 0.45-μm-pore-size polyvinylidene difluoride (PVDF) membranes (Millipore). The membranes were incubated overnight in blocking solution (1× PBS, pH 7.4, 0.01% Tween 20, 5% milk powder), after which proteins were detected with 1:7,000-diluted and 1:10,000-diluted rabbit anti-mouse M2-specific antiserum (DakoCytomation). Bound antibodies were visualized using ECL chemiluminescent detection reagent (PerkinElmer).

## RESULTS

### The RsmA-dependent diguanylate cyclase PA0338 is not involved in the *retS* mutant hyperbiofilm phenotype.

**FIG 1:**
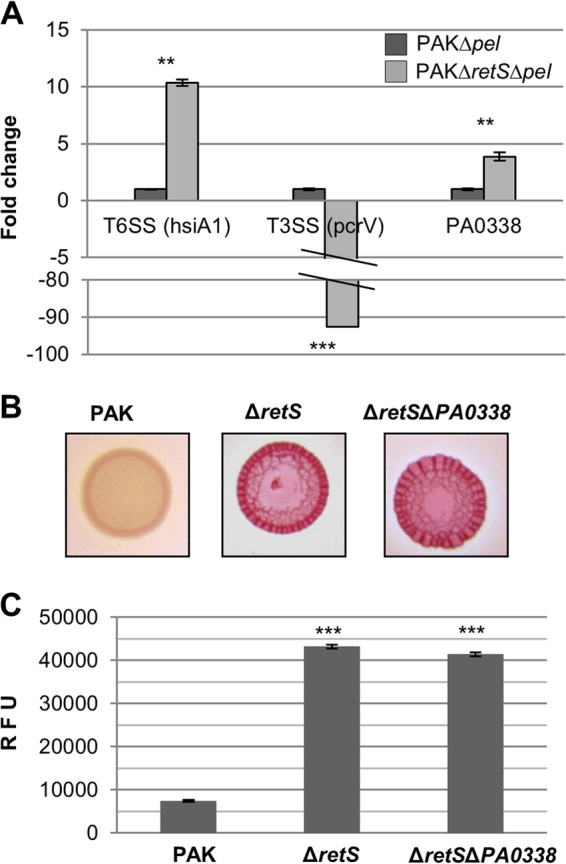
PA0338 is upregulated in a *retS* mutant background. (A) Transcript levels of *hsiA1* (positive control), *pcrV* (negative control), and *PA0338* measured by qRT-PCR and normalized to those of *gyrA*. Statistical Student's *t*-test analysis was based on three replicates, and significant changes are indicated (**, *P* < 0.001; ***, *P* < 0.0001). (B) Congo red binding of the indicated strains after 2 days of incubation. (C) Intracellular levels of c-di-GMP measured with the transcriptional P*cdrA-gfp* reporter. Relative fluorescence units (RFU) are arbitrary fluorescence intensity units corrected for cell density. At least three independent experiments were performed. Statistically significant changes were calculated using the Student *t* test and are indicated (***, *P* < 0.0001).

It has previously been shown that in a P. aeruginosa PAK *retS* mutant, the levels of c-di-GMP are increased (30). We hypothesized that this could be a consequence of either (i) an upregulation or activation of proteins with DGC activity or (ii) a downregulation or inactivation of proteins with PDE activity. Since a *retS* mutation results in the relief of RsmA repression, the RsmA regulon (22) was screened for putative upregulated DGCs or downregulated PDEs. Analysis of the data published by Brencic and Lory in 2009 (22) revealed that only one putative DGC, PA0338, was upregulated (2.8-fold) in the *rsmA* mutant compared to its level of regulation in the PAK wild-type strain, and no putative PDE was downregulated. Using qRT-PCR, we were able to show that PA0338 mRNA levels are 3.8-fold higher in a *retS* background than in the parental PAK strain (Fig. 1A). RetS is also known to inversely regulate the type III secretion system (T3SS) and type VI secretion system (T6SS) via RsmA (30), and our qRT-PCR confirmed this observation (Fig. 1A). In light of the *PA0338* upregulation, we tested whether a mutation in *PA0338* resulted in the suppression of the *retS* hyperbiofilm phenotype. A *retS PA0338* mutant was engineered and spotted on Congo red agar plates. Both the *retS* and *retS PA0338* mutants displayed a wrinkly red phenotype that contrasts with the smooth white appearance of the PAK wild-type strain, suggesting that the double mutant is still a hyperbiofilm former (Fig. 1B). The level of c-di-GMP was also monitored in these strains using the P*cdrA-gfp* reporter fusion (33). As shown in Fig. 1C, the *retS* and *retS PA0338* mutants displayed elevated levels of c-di-GMP. Therefore, these data suggest that PA0338 is not involved in the synthesis of c-di-GMP that leads to the hyperbiofilm phenotype of the *retS* mutant.

### SadC is responsible for the *retS* mutant hyperbiofilm phenotype.

**FIG 2:**
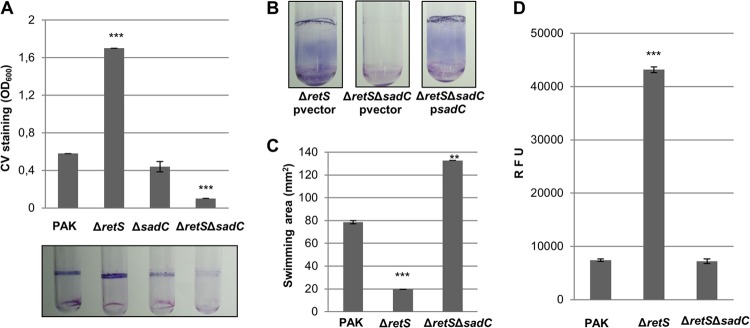
SadC is required for the *retS* mutant hyperbiofilm phenotype. (A) Quantification of the crystal violet staining of biofilms grown in microtiter plates for 14 h. A photo of the test tubes was taken prior to the addition of ethanol for quantification purposes. (B) Crystal violet staining of biofilms grown in test tubes for 6 h. The empty vector pBBR1MCS-4 (pvector) and pBBR1MCS-4-*sadC* (p*sadC*) were conjugated into PAK Δ*retS* or PAK Δ*retS* Δ*sadC*. (C) Results of a swimming motility assay performed in 0.3% LB agar plates. (D) Intracellular levels of c-di-GMP measured with the transcriptional P*cdrA-gfp* reporter. Relative fluorescence units (RFU) are arbitrary fluorescence intensity units corrected for cell density. At least three independent experiments were performed. (A, C, and D) Statistically significant changes were calculated using the Student *t* test and are indicated (**, *P* < 0.001; ***, *P* < 0.0001).

In the initial characterization of the *retS* mutant (15), a transposon mutagenesis screen for suppressors of the *retS* hyperbiofilm phenotype was performed, and insertions were mapped to the *gacS*, *gacA*, *rsmZ*, and *PA4332* (*sadC*) genes. SadC is a membrane protein and has a cytoplasmic C-terminal GGDEF domain with known DGC activity (31, 35). Herein, we engineered a *retS sadC* mutant and examined the biofilm phenotype using crystal violet staining. As a control, a *sadC* single mutant was also included. Interestingly, deletion of *sadC* in the *retS* background readily resulted in the loss of the *retS* mutant hyperbiofilm phenotype (Fig. 2A). This loss of phenotype was more pronounced than the loss caused by the deletion of *sadC* alone (Fig. 2A) and could be complemented by introducing the *sadC* gene in *trans* (Fig. 2B). Moreover, the reduced ability of the *retS sadC* mutant to form biofilms was accompanied by an increase in the ability of the strain to swim in soft agar plates (Fig. 2C). Finally, the *retS sadC* mutant displayed levels of c-di-GMP similar to those displayed by the wild-type strain (Fig. 2D), suggesting that the SadC diguanylate cyclase is active in the *retS* mutant and is responsible for the hyperbiofilm phenotype.

### SadC is responsible for the *hptB* mutant hyperbiofilm phenotype.

**FIG 3:**
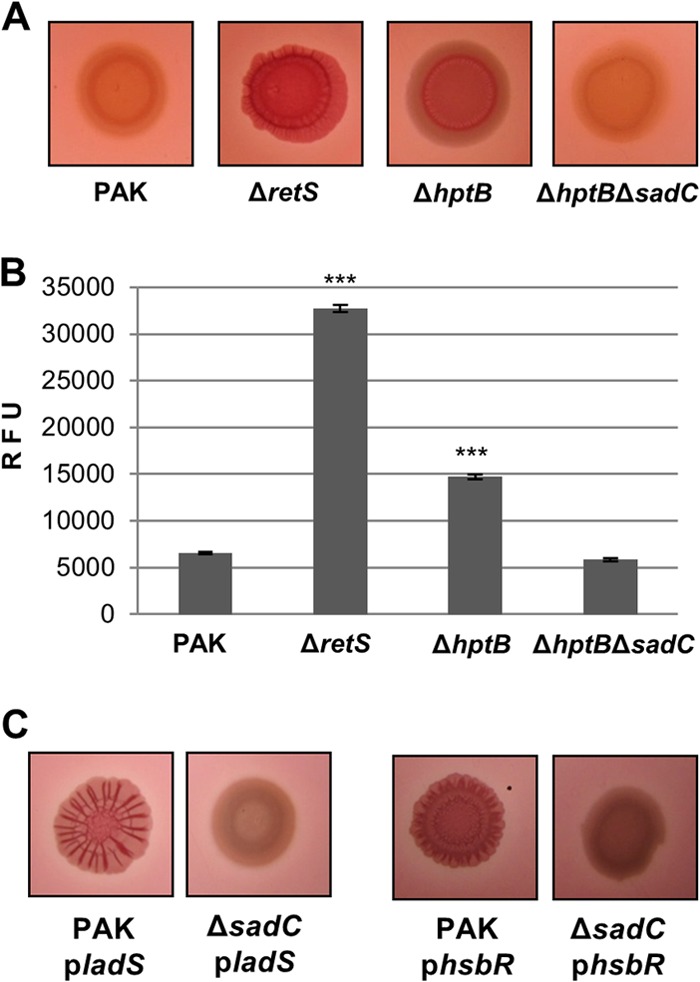
SadC is required for the *hptB* mutant hyperbiofilm phenotype. (A) Congo red binding of the indicated strains after 2 days of incubation. (B) Intracellular levels of c-di-GMP measured with the transcriptional P*cdrA-gfp* reporter. Relative fluorescence units (RFU) are arbitrary fluorescence intensity units corrected for cell density. At least three independent experiments were performed. Statistically significant changes are indicated (***, *P* < 0.0001, Student's *t* test). (C) PAK and PAK Δ*sadC* were conjugated with pBBR1-MCS4-*ladS* (p*ladS*) or pBBR1-MCS4-*hsbR* (p*hsbR*). Congo red binding is from day 2 of incubation.

We next investigated the role of SadC in a known regulatory pathway that converges onto the Gac/Rsm pathway and involves the histidine phosphotransfer module HptB (36–38). It was shown previously that an *hptB* mutant displays a hyperbiofilm phenotype and this phenotype is milder than the one observed for the *retS* mutant (36). Analysis of the c-di-GMP levels and Congo red binding showed that the intermediate level of biofilm formation of the *hptB* mutant corresponds to an intermediate level of intracellular c-di-GMP, and both phenotypes were abrogated in an *hptB sadC* mutant (Fig. 3A and B). To further establish the pivotal role of SadC in these RsmA-dependent pathways, an inverse approach was used whereby the RetS and HptB antagonists, namely, LadS (17) and HsbR (36, 39), respectively, were overexpressed in a *sadC* mutant. For this purpose, plasmids encoding either the sensor LadS or the response regulator HsbR were introduced by conjugation into the relevant strains. While overexpression of *ladS* and *hsbR* in the wild-type strain induced Congo red binding, a similar phenotype could not be obtained in the *sadC* mutant (Fig. 3C). Overall, these data suggest that the increase of c-di-GMP levels and the induction of biofilm-related phenotypes observed upon activation of the Gac/Rsm cascade rely entirely on the diguanylate cyclase SadC.

### SadC production is controlled by RsmA.

**FIG 4:**
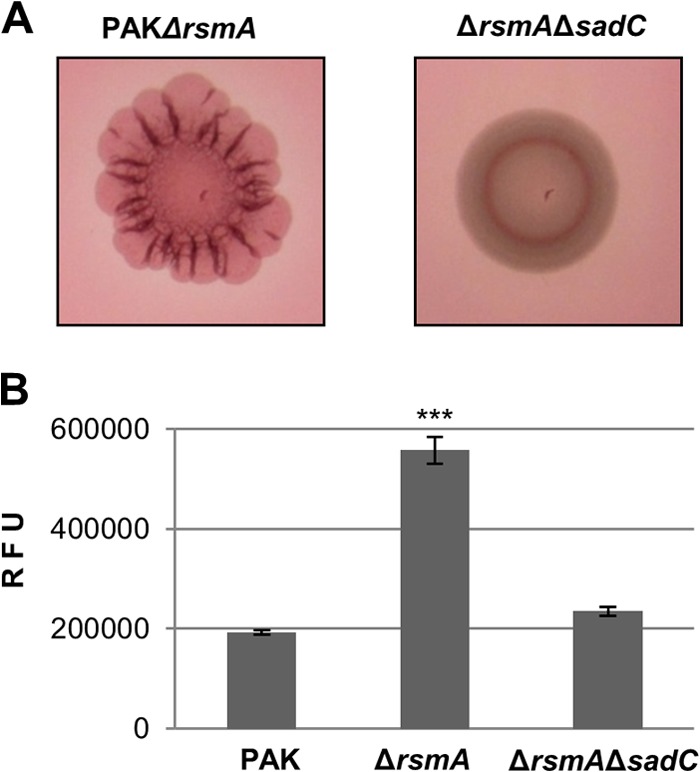
Deletion of *sadC* abrogates the hyperbiofilm and high c-di-GMP phenotypes of an *rsmA* mutant. (A) Congo red binding phenotypes visualized on day 2 of incubation. (B) Intracellular levels of c-di-GMP measured with the transcriptional P*cdrA-gfp* reporter. Relative fluorescence units (RFU) are arbitrary fluorescence intensity units corrected for cell density. Statistically significant changes are indicated (***, *P* < 0.0001, Student's *t* test).

**FIG 5:**
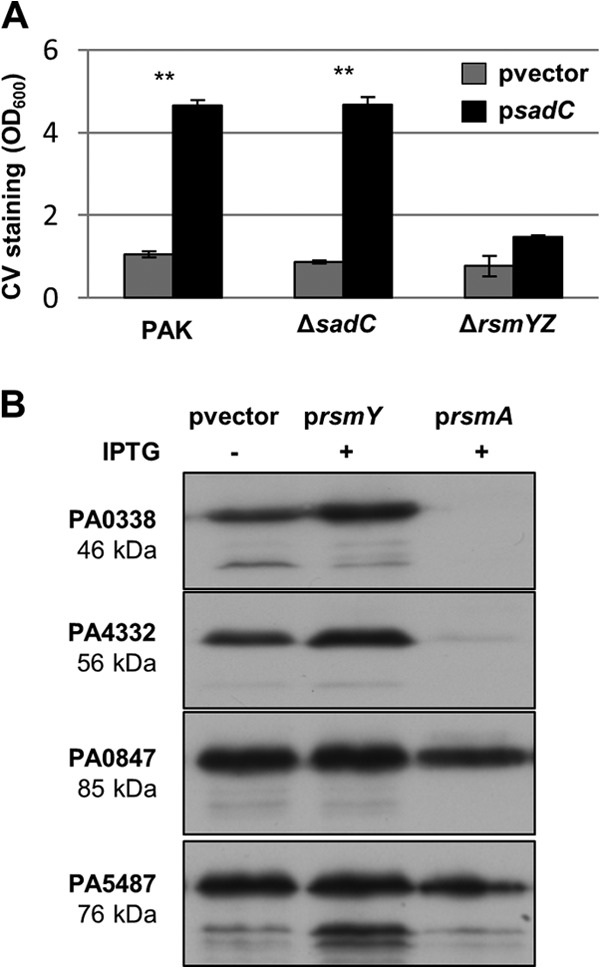
SadC production is controlled by RsmA. (A) Quantification of the crystal violet staining of biofilms grown in microtiter plates for 6 h. Statistically significant changes are indicated (**, *P* < 0.001, Student's *t* test). (B) Immunoblots with M2 antiserum showing the levels of DGC-Flag variants in whole-cell lysates. The PAO1 DGC-Flag strains indicated on the left contain different plasmids: pvector (empty vector), p*rsmY* (IPTG-inducible *rsmY*), and p*rsmA* (IPTG-inducible *rsmA*). Cells were harvested after 1 h induction with 1 mM IPTG. Shown here is a representative immunoblot from at least three independent experiments.

Both the RetS- and HptB-dependent pathways ultimately relay onto the translational repressor RsmA, and therefore, the *sadC* deletion was also engineered in an *rsmA* mutant background. Note that the *rsmA* mutant displayed a wrinkly red phenotype and this corresponded to an increased level of c-di-GMP (Fig. 4A and B). As was observed for the *retS sadC* and *hptB sadC* double mutants, hyperbiofilm formation and the increase in c-di-GMP levels were also lost in the *rsmA sadC* mutant (Fig. 4A and B). In addition, a crystal violet assay showed that the overexpression of *sadC* resulted in hyperbiofilm formation in the wild-type or *sadC* mutant strains but failed to do so in the *rsmYZ* mutant (Fig. 5A). This suggests that *sadC* overexpression cannot relieve the repression by RsmA and is in favor of a link between these two signaling pathways, yet it is a possibility that the lack of biofilm is due to the absence of *rsmYZ*, a reduced level of c-di-GMP, or both. In order to address this further, we then used an alternative strategy. As discussed above, previous studies identified PA0338 as a direct target for RsmA (22), but no RsmA-dependent control on SadC production was reported. Here, we used an original approach to look at the production of SadC in a genetic background where either *rsmA* or one of its regulatory antagonists, the small RNA *rsmY*, is overexpressed. To do this, the chromosomally encoded *sadC* was genetically modified to encode a FLAG-tagged version of the protein, and the levels of SadC were monitored by Western blotting using antibodies against the tag. Strikingly, SadC was readily detected when the RsmA repression was released by RsmY overproduction, whereas SadC production was drastically reduced upon RsmA induction (Fig. 5B). The same was observed for PA0338 but not for two additional DGCs that were taken as negative controls, namely, PA0847 and PA5487 (Fig. 5B).

### SadB is also central to the Gac/Rsm pathway.

**FIG 6:**
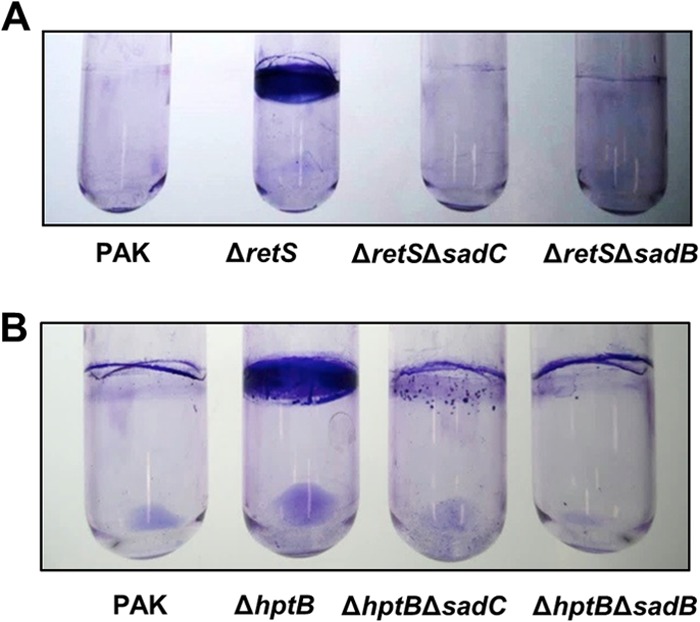
SadB is central to the RetS- and HptB-dependent signaling pathways. Crystal violet staining of biofilms grown under shaking conditions for 8 h (A) and 18 h (B) is shown.

Previous work (31) has placed SadB and SadC in the same genetic pathway regulating biofilm formation. Although SadB is a protein of unknown function (40), it was shown that it is required for SadC signaling. We thus tested whether a *sadB* mutation was able to suppress the phenotype associated with *retS* or *hptB* mutations, as was observed for a *sadC* mutation. In both cases, the hyperbiofilm phenotype was significantly reduced upon introduction of the *sadB* deletion (Fig. 6). Altogether, our results indicate that the Gac/Rsm pathway is tightly linked to c-di-GMP signaling via the SadC/SadB pathway.

## DISCUSSION

**FIG 7:**
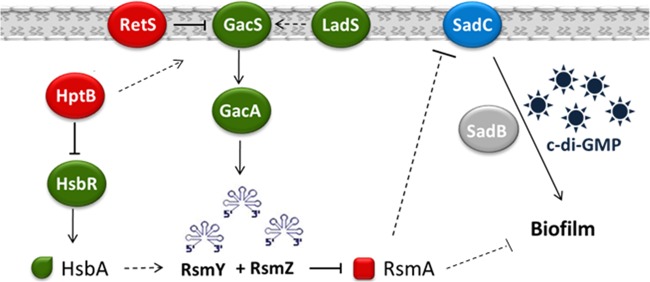
The SadC and the Gac/Rsm pathways are interlinked. The Gac/Rsm system is a complex signaling cascade that regulates several biological functions, including biofilm formation. The proteins belonging to this cascade that negatively impact the formation of biofilms are indicated in red. All the proteins of this cascade indicated in green have a positive impact on biofilm formation. The hyperbiofilm phenotype induced by the deletion of the *retS*, *hptB*, or *rsmA* gene is dependent on an intact *sadC* gene product to produce c-di-GMP (represented by stars). SadC levels are under the control of RsmA, but the exact mechanism by which it occurs is still unknown. Downstream of SadC is SadB, whose function remains obscure.

In P. aeruginosa there are more than 40 putative proteins involved in the metabolism of c-di-GMP, and this is thought to form an intricate intracellular network that specifically regulates biological processes crucial for bacterial adaptation and virulence (41). However, many of the studies about this second messenger have used genetic approaches whereby a particular DGC or PDE is overexpressed, and this seems to have a general impact in dictating the lifestyle fate of a bacterial population, failing to establish a more specific function. Here, we sought to identify the protein or proteins specifically responsible for the elevated levels of c-di-GMP in a *retS* mutant, thus physically connecting the two networks (Fig. 7). RetS is a sensor protein that represses Gac/Rsm signaling by forming heterodimers with the GacS sensor (15). Although the complexity of the Gac/Rsm system varies between different species, the GacA/GacS two-component system, the cognate small RNA targets and the translational repressor, are conserved in Gammaproteobacteria (24). In some species, a specific link between the Gac/Rsm pathway and c-di-GMP signaling has been reported in the last few years. In E. coli, the GacA/GacS two-component system is known as BarA/UvrY and controls the expression of the small RNAs CsrB and CsrC. These, in turn, modulate the activity of the CsrA translational repressor, which targets, among many other genes, two genes encoding proteins with a GGDEF domain, YdeH and YcdT (42). In Salmonella enterica, the GacA/GacS two-component system is known as BarA/SirA and modulates the CsrA translational repressor via CsrB and CsrC. In this case, CsrA is known to regulate eight genes encoding GGDEF, GGDEF/EAL, or EAL domain proteins, and five of these are regulated by direct binding of CsrA to the mRNA (43). In Xanthomonas campestris, RsmA has been shown to control posttranscriptionally at least three GGDEF domain proteins, and the three contribute additively to the elevated levels of c-di-GMP in the *rsmA* mutant (44). In P. aeruginosa, direct evidence that the Gac system and c-di-GMP signaling were interlinked came from the observation that the *retS* mutant displays high levels of c-di-GMP and that the c-di-GMP-induced T3SS/T6SS switch is dependent on the two sRNAs RsmY and RsmZ (30).

On the basis of the published literature, two proteins with a GGDEF domain were considered of interest in this study. On the one hand, *PA0338* was reported to be upregulated in an *rsmA* mutant (22), and on the other hand, a *PA4332* (*sadC*) transposon mutant had been identified as a suppressor of the *retS* mutant hyperbiofilm phenotype (15). By engineering mutants with deletions of these genes, we were able to conclude that the activity of SadC is directly responsible for the hyperbiofilm phenotype and the elevated levels of c-di-GMP observed in a *retS* mutant. To further understand the importance of SadC in the Gac/Rsm pathway, deletion of *sadC* was also introduced into *hptB* and *rsmA* mutant backgrounds. The HptB pathway is known to converge onto RsmA, possibly via the transcriptional repression of RsmY (36). In addition, HptB can be phosphorylated by the sensor PA1611 (37), which is able to interact with RetS to counteract its repressing role upon GacS (21). Once it is phosphorylated, HptB phosphorylates HsbR, whose output domain has both a phosphatase activity and a kinase activity (36, 45). HsbR controls the phosphorylation state of HsbA, an anti-anti-sigma factor that is likely to control the availability of a sigma factor required for *rsmY* expression. For the flagellum biogenesis, it has recently been shown that this anti-anti-sigma factor regulates the anti-sigma factor FlgM, releasing σ^28^ (45). In both the *htpB sadC* and *rsmA sadC* mutants, we observed the abrogation of the hyperbiofilm phenotype and a restoration of the c-di-GMP levels.

Even though PA0338 does not seem to account for the production of c-di-GMP in the *retS* mutant, both qRT-PCR and Western blot analysis indicate that the expression and production of PA0338 are under the control of RsmA. This may suggest that the catalytic activity of PA0338 is too low to be a major contributor to biofilm formation or that it might be connected to other RsmA-dependent phenotypes, yet it was previously shown that overexpression of PA0338 is able to increase the biofilm levels in PA14 (46), a genuine *ladS* mutant (47). In the case of SadC, RsmA control was detected only at the level of protein production, while no regulation of transcript abundance was observed by qRT-PCR (data not shown).

SadC was previously characterized for its role in swarming motility and put in the same genetic pathway as BifA (48), a PDE, and SadB, a protein of unknown function (40). In addition, RoeA, a DGC, has been shown to have an additive effect with SadC in P. aeruginosa strain PA14 (35, 49). Since SadB has been demonstrated to act downstream of SadC, the impact of a *sadB* deletion in the *retS* and *hptB* mutants was also investigated, and the results confirmed the position of SadB downstream of SadC in the signaling cascade, although the function of SadB is not clear. It has previously been shown that both the N-terminal YbaK domain of SadB and the C-terminal HD domain of SadB are required for function (40, 50). Hypothetically, it was considered that SadB could act upstream of c-di-GMP synthesis, somehow assisting SadC to achieve its DGC activity, or that SadB could function as a c-di-GMP receptor, somehow transmitting the c-di-GMP signal to downstream targets. In addition to this, SadB is known to act upstream of the Pil-Chp chemotactic cluster (50), and this cluster is required for type IV pilus (T4P) biogenesis (51). Interestingly, in Pseudomonas fluorescens, a link between SadB and the Gac system in which it exerts a negative regulation on flagellum-driven motility during exponential phase has recently been established (52). In this bacterium, the Gac system comprises three translational repressors, RsmA, RsmE, and RsmI (53), and the two signaling cascades were shown to intersect in the cooperative regulation of the σ^22^ sigma factor, also known as AlgT or AlgU. On the one hand, SadB is required for the transcription of σ^22^, and on the other hand, RsmA and RsmE act as translational repressors of σ^22^. Once it is produced, σ^22^ is necessary for the expression of the transcriptional regulator *amrZ* (also referred to as *algZ*) (54, 55), which functions to downregulate *fleQ*, a c-di-GMP binding protein (56) and the master regulator of the flagellar components. In P. aeruginosa, it is not known if σ^22^ is a member of the RsmA regulon, but AmrZ belongs to the σ^22^ regulon and has been shown to be involved in both the downregulation of flagella and Psl exopolysaccharide and the upregulation of twitching motility and alginate production (57–60).

In conclusion, the regulatory pathways involved in the control of P. aeruginosa lifestyles are increasingly complex. The connection between Gac/Rsm signaling and c-di-GMP is unlikely to be restricted to biofilm control or the T3SS/T6SS switch, and recent studies have also highlighted that iron uptake is coordinately regulated by these two networks (61). It is thus important to study in further depth the direct connections existing between each component and preferably avoid an overexpression context. Our future work will aim at establishing what the downstream targets of c-di-GMP signaling via SadC are. More particularly, we will investigate what the function of SadB is, although it is likely that other important players in the cascade are still to be identified.

## Supplementary Material

Supplemental material
