# Supplementary material for: The Diguanylate Cyclase SadC Is a Central Player in Gac/Rsm-Mediated Biofilm Formation in Pseudomonas aeruginosa
Source: J Bacteriol. 2014 Dec;196(23):4081–8. doi: 10.1128/JB.01850-14 (PMC4248864; doi:10.1128/JB.01850-14)
Supplement: Supplemental material [file supp_196_23_4081__index.html]

Supplemental material 

# The Diguanylate Cyclase SadC Is a Central Player in Gac/Rsm-Mediated Biofilm Formation in Pseudomonas aeruginosa

## Supplemental material

**Files in this Data Supplement:**

- Supplemental file 1 -

  Table S1 (Strains, plasmids, and primers)

  PDF, 171K
