# Supplementary material for: The Diguanylate Cyclase SadC Is a Central Player in Gac/Rsm-Mediated Biofilm Formation in Pseudomonas aeruginosa
Source: J Bacteriol. 2014 Dec;196(23):4081–8. doi: 10.1128/JB.01850-14 (PMC4248864; doi:10.1128/JB.01850-14)
Supplement: Supplemental material [file JB.01850-14_zjb999093379so1.pdf]

Supplementary material

Table S1. Strains, plasmids and primers used in this study.

Strain/Plasmid/Primer	Relevant characteristics[a]	Source
Escherichia coli		
TOP10	F^- *mcr*A Δ(*mrr-hsd*RMS-*mcr*BC) φ80*lac*ZΔM15 Δ*lac*X74 *rec*A1 *ara*D139 Δ(*ara-leu*) 7697 *gal*U *gal*K *rps*L (Str^R) *end*A1 *nup*G λ^-	Invitrogen
OmniMAX	F′ {*pro*AB+ *lac*I^q *lac*ZΔM15 Tn10(Tet^R) Δ(*ccd*AB)} *mcr*A Δ(*mrr-hsd*RMS-*mcr*BC) Φ80*lac*ZΔM15 Δ(*lac*ZYA-*arg*F) U169 *end*A1 *rec*A1 *sup*E44 *thi*-1 *gyr*A96 *rel*A1 *ton*A *pan*D	Invitrogen
CC118λpir	Host strain for pKNG101; D (*ara-leu*) *araD DlacX74 galE galK-phoA20 thi-1 rpsE rpoB argE* (Am) *recA1 Rf*^{r} *(lpir)*	(1)
1047	Helper strain for conjugation of plasmids harbouring pRK2013, Km^R	Lab collection
S17-1	F^-, lambda (-), *endA, thi, pro,recA*, restriction (-) modification (+), RP4 derivative integrated into the chromosome with *Tet::Mu, Km::Tn7*	Lab collection
Pseudomonas aeruginosa		
PAK	Wild-type	(2)
PAKΔ*retS*	PAK with a *retS* deletion	(2)
PAKΔ*pelG*	PAK with a *pelG* deletion	(3)
PAKΔ*retS*Δ*pelF*	PAK with deletions of *retS* and *pelF*	(4)
PAKΔ*retS*Δ*PA0338*	PAK with deletions of *retS* and *PA0338*	This study
PAKΔ*retS*Δ*sadC*	PAK with deletions of *retS* and *sadC*	This study
PAKΔ*sadC*	PAK with a *sadC* deletion	This study
PAKΔ*retS*Δ*sadB*	PAK with deletions of *retS* and *sadB*	This study
PAKΔ*retS*Δ*sadC*Δ*bifA*	PAK with deletions of *retS, sadC* and *bifA*	This study
PAKΔ*hptB*	PAK with a *hptB* deletion	(5)
PAKΔ*hptB*Δ*sadC*	PAK with deletions of *hptB* and *sadC*	This study
PAKΔ*hptB*Δ*sadB*	PAK with deletions of *hptB* and *sadB*	This study
PAKΔ*rsmA*	PAK with a *rsmA* deletion	(6)
PAKΔ*rsmA*Δ*sadC*	PAK with deletions of *rsmA* and *sadC*	This study
PAKΔ*rsmYZ*	PAK with deletions of *rsmY* and *rsmZ*	(5)
PA01	Wild-type	Lab collection
PA01 PA0338-Flag	PA01 containing PA0338 with a chromosomal C-terminal Flag-tag	This study
PA01 PA0847-Flag	PA01 containing PA0847 with a chromosomal C-terminal Flag-tag	This study
PA01 PA4332-Flag	PA01 containing PA4332 with a chromosomal C-terminal Flag-tag	This study
PA01 PA5487-Flag	PA01 containing PA5487 with a chromosomal C-terminal Flag-tag	This study
Plasmids		
pCR2.1-TA	Cloning plasmid, Ap^R, Km^R	Invitrogen
pBBR1MCS-4	Broad host range vector, Ap^R	(7)
pBBR1MCS-4-*sadC*	*sadC* cloned into pBBR1MCS-4 (ApaI/BamHI)	This study
pBBR1MCS-4-*ladS*	*ladS* cloned into pBBR1MCS-4	(6)
pBBR1MCS-4-*hsbR*	*hsbR* cloned into pBBR1MCS-4	(5)

Strain/Plasmid/Primer	Relevant characteristics[a]	Source
pKNG101	Suicide vector carrying *sacB*, *SmR*	(8)
pKNG101-Δ*PA0338*	Mutator fragment for deletion of PA0338 (from amino acid 2 to 367), Sm^R	This study
pKNG101-Δ*sadC*	Mutator fragment for deletion of *sadC* (from amino acid 87 to 414), Sm^R	This study
pKNG101-Δ*sadB*	Mutator fragment for deletion of *sadB* (from amino acid 4 to 467), Sm^R	This study
pKNG101-Δ*bifA*	Mutator fragment for deletion of *bifA* (from amino acid 4 to 657), Sm^R	This study
P_{cdrA}-*gfp*	Plasmid expressing *gfp* from the promoter of *cdrA*, Ap^R, Gm^R	(9)
pME3087	Tet^R, suicide vector for allelic replacement; *ColE1*-replicon, *IncP-1*, *Mob*	(10)
pME3087-0338-Flag	pME3087 with the cassette for introducing a c-terminal Flag-tag on PA0338 (BamHI/EcoRI)	This study
pME3087-0847-Flag	pME3087 with the cassette for introducing a c-terminal Flag-tag on PA0847 (BamHI/HinDIII)	This study
pME3087-4332-Flag	pME3087 with the cassette for introducing a c-terminal Flag-tag on PA4332 (BamHI/HinDIII)	This study
pME3087-5487-Flag	pME3087 with the cassette for introducing a c-terminal Flag-tag on PA5487 (BamHI/EcoRI)	This study
pME6918	Tet^R, *rsmY* under Ptac promoter at position +1 in pME6032	(11)
pME3849	Gm^R, *rsmA* under Plac promoter in pME6001 (BamHI/SmaI)	(12)
Primers for qRT-PCR		
qGyrAF	AACGACTGGAACAAGCCCTACA	This study
qGyrAR	GCGCACGATGGTGTCGTA	This study
qPcrVF	AGTGGGATCTGCGCGAGTT	This study
qPcrVR	TGGGTCTGCAGGACATCCTT	This study
qHsiA1F	ATCGCCTGCTCGAGTATTACG	This study
qHsiA1R	GGGATGAGATTCCTCACGATTT	This study
qPA0338F	GAGCGTGGAGACCTGGAAGA	This study
qPA0338R	GCAGTATTCCGGGCTTTCG	This study
Primers for deletion mutants		
1PA0338F	GACTTCCTTTCGGTCGGTTC	This study
2PA0338R	ATCGCGACGCACGACGAAGCGTCC	This study
3PA0338F	TTCGTCGTGCGTCGCGATTGCGTG	This study
4PA0338R	CCATCAGCTCTGGCACTACA	This study
5PA0338F	ATCCTGCTGCCGATGATCT	This study
6PA0338R	ACTATTCCGGCTTCAACCTG	This study
1SadCF	TATCGTCGACGCTCATGGTA	This study
2SadCR	TTCCAGCTGAGCGGACCGCGGATTTATC	This study
3SadCF	CGGTCCGCTCAGCTGGAAAGCTGTTGCGA	This study
4SadCR	TCAGGATCGAAAGGCTGCAA	This study
5SadCF	ATGGTCGGTACGGTTTTCAG	This study
6SadCR	CTCCACAGCAGCAGGTGCT	This study
1SadBF	ACTGCGTGCTGCTCTACCAC	This study
2SadBR	TCACCCCGGTTCTGTCATGACGAGACCATG	This study

Strain/Plasmid/Primer	Relevant characteristics[a]	Source
3SadBF	ATGACAGAACCGGGGTGACCGGGTAG	This study
4SadBR	GGCAAGGACCTGGTGTTCCAGTT	This study
5SadBF	CAACGCCAGCCTGATGAT	This study
6SadBR	CGTCTTCTCCTGTGGATGCT	This study
1BifAF	GCTGGCTGGTGAAGAAGG	This study
2BifAR	GCTGTACAGCAGTTTCAAGGGGCCTTCCT	This study
3BifAF	TTGAAACTGCTGTACAGCAAGCCGCTGC	This study
4BifAR	GTCGCCAGGGACTCGATG	This study
5BifAF	GGCTCCTTCGACAAGTTCAA	This study
6BifAR	CTCTACCGCTACACCGAGGA	This study
Primers for cloning of sadC		
sadCF	TTCACACAGGAAAAGGAGATGAACTGCAGGGC	This study
sadCR	GATCGAAAGGCTGCAACAC	This study
Primers introduction of Flag-tag		
PA0338Flag up F BamH1	GACCTGGAAGAGGATCCTCCATCC	This study
PA0338Flag up R	CTATTTATCGTCGTCATCTTTGTAGTCGATATCATGATCTTT ATAATCACCGTCATGGTCTTTGTAGTCGAAGCAACAGG CCACGCAATCGC	This study
PA0338Flag down F	GACTACAAAGACCATGACGGTGATTATAAAGATCATGATA TCGACTACAAAGATGACGACGATAAATAGTAAAGGTC AGCCTGGCGGCAGCACCAG	This study
PA0338Flag down R EcoRI	CCAACCGTGACGAATTCTATGCC	This study
PA0847Flag up F BamHI	CCATCCCTACTAGGATCCGCTC	This study
PA0847Flag up R	CTATTTATCGTCGTCATCTTTGTAGTCGATATCATGATCTTT ATAATCACCGTCATGGTCTTTGTAGTCGAAGGCTGGCG CCTGGTACAGGCAATAG	This study
PA0847Flag down F	GACTACAAAGACCATGACGGTGATTATAAAGATCATGATA TCGACTACAAAGATGACGACGATAAATAGTAAAATGCGCG GACAAACAGG	This study
PA0847Flag down R HinDIII	CATCCAGATGCCGAAAAGCTTCCACAG	This study
PA4332Flag up F BamHI	CGTTCATCGCTTTTTCCGGATCCTACCTG	This study
PA4332Flag up R	CTATTTATCGTCGTCATCTTTGTAGTCGATATCATGATCTTT ATAATCACCGTCATGGTCTTTGTAGTCGAAGGCACTGG TGACCTCCCAGG	This study
PA4332 Flag down F	GACTACAAAGACCATGACGGTGATTATAAAGATCATGATA TCGACTACAAAGATGACGACGATAAATAGTAAGTGCCTGA CATACGGGTCGG	This study
PA4332Flag down R HinDIII	CAGGTGAAGCTTCTGGATCG	This study
PA5487Flag up F BamHI	GTCAGTGGCCTGGATCCCAGCGTGC	This study
PA5487Flag up R	CTATTTATCGTCGTCATCTTTGTAGTCGATATCATGATCTTT ATAATCACCGTCATGGTCTTTGTAGTCGAAGGCCACTT CCAGGCGGTCCC	This study
PA5487Flag down F	GACTACAAAGACCATGACGGTGATTATAAAGATCATGATA	This study

Strain/Plasmid/Primer	Relevant characteristics[a]	Source
	TCGACTACAAAGATGACGACGATAAATAGTAAAGGGCGAC CAGCGCACATTC	
PA5487Flag down R EcoRI	CGAGGGAATTCTGGAGGAAC	This study

[a]Ap^R, Ampicillin resistance; Str^R, Streptomycin resistance; Km^R, Kanamycin resistance; Gm^R, Gentamicin resistance; Tet^R, Tetracyclin resistance.

References

1. **Herrero M, de Lorenzo V, Timmis KN.** 1990. Transposon vectors containing non-antibiotic resistance selection markers for cloning and stable chromosomal insertion of foreign genes in gram-negative bacteria. J Bacteriol **172:**6557-6567.
2. **Goodman AL, Merighi M, Hyodo M, Ventre I, Filloux A, Lory S.** 2009. Direct interaction between sensor kinase proteins mediates acute and chronic disease phenotypes in a bacterial pathogen. Genes Dev **23:**249-259.
3. **Vasseur P, Vallet-Gely I, Soscia C, Genin S, Filloux A.** 2005. The pel genes of the Pseudomonas aeruginosa PAK strain are involved at early and late stages of biofilm formation. Microbiology **151:**985-997.
4. **Sadovskaya I, Vinogradov E, Li J, Hachani A, Kowalska K, Filloux A.** 2010. High-level antibiotic resistance in Pseudomonas aeruginosa biofilm: the ndvB gene is involved in the production of highly glycerol-phosphorylated beta-(1->3)-glucans, which bind aminoglycosides. Glycobiology **20:**895-904.
5. **Bordi C, Lamy MC, Ventre I, Termine E, Hachani A, Fillet S, Roche B, Bleves S, Mejean V, Lazdunski A, Filloux A.** 2010. Regulatory RNAs and the HptB/RetS signalling pathways fine-tune Pseudomonas aeruginosa pathogenesis. Mol Microbiol **76:**1427-1443.
6. **Ventre I, Goodman AL, Vallet-Gely I, Vasseur P, Soscia C, Molin S, Bleves S, Lazdunski A, Lory S, Filloux A.** 2006. Multiple sensors control reciprocal expression of Pseudomonas aeruginosa regulatory RNA and virulence genes. Proc Natl Acad Sci U S A **103:**171-176.
7. **Kovach ME, Elzer PH, Hill DS, Robertson GT, Farris MA, Roop RM, 2nd, Peterson KM.** 1995. Four new derivatives of the broad-host-range cloning vector pBBR1MCS, carrying different antibiotic-resistance cassettes. Gene **166:**175-176.
8. **Kaniga K, Delor I, Cornelis GR.** 1991. A wide-host-range suicide vector for improving reverse genetics in gram-negative bacteria: inactivation of the blaA gene of Yersinia enterocolitica. Gene **109:**137-141.
9. **Rybtke MT, Borlee BR, Murakami K, Irie Y, Hentzer M, Nielsen TE, Givskov M, Parsek MR, Tolker-Nielsen T.** 2012. Fluorescence-based reporter for gauging cyclic di-GMP levels in Pseudomonas aeruginosa. Appl Environ Microbiol **78:**5060-5069.
10. **Voisard C, Bull CT, Keel C, Laville J, Maurhofer M, Schnider U, Defago G, Haas D.** 1994. Biocontrol of Root Diseases by Pseudomonas-Fluorescens Cha0 -

Current Concepts and Experimental Approaches. Molecular Ecology of Rhizosphere Microorganisms**:**67-89.

11. **Valverde C, Heeb S, Keel C, Haas D.** 2003. RsmY, a small regulatory RNA, is required in concert with RsmZ for GacA-dependent expression of biocontrol traits in Pseudomonas fluorescens CHA0. Mol Microbiol **50:**1361-1379.
12. **Pessi G, Williams F, Hindle Z, Heurlier K, Holden MT, Camara M, Haas D, Williams P.** 2001. The global posttranscriptional regulator RsmA modulates production of virulence determinants and N-acylhomoserine lactones in Pseudomonas aeruginosa. J Bacteriol **183:**6676-6683.
